# Radioimmunotherapy With WT1 Dendritic Cell Vaccine for End-Stage Lung Adenocarcinoma Markedly Shrinks Tumors

**DOI:** 10.7759/cureus.48412

**Published:** 2023-11-06

**Authors:** Hisashi Nagai, Ryusuke Karube, Fengxiang Zhao

**Affiliations:** 1 Human and Environmental Studies, Tokai University, Kanagawa, JPN; 2 Oncology, Ginza Phoenix Clinic, Tokyo, JPN

**Keywords:** metastatic non-small cell lung cancer, cancer immunotherapy, immunoradiation therapy, radioimmunotherapy, dendritic cell

## Abstract

In advanced lung adenocarcinoma with metastases, the current standard of care does not in principle include aggressive cancer treatment with surgery and radiotherapy. Therefore, when chemotherapy cannot be continued, the patient is generally switched to palliative care. Our patient with stage IV lung adenocarcinoma in his 60s was receiving chemotherapy, which had to be discontinued due to severe side effects. As standard treatment was no longer indicated, he underwent radioimmunotherapy combined with WT1 dendritic cell vaccine therapy. As a result, the massive lung cancer shrank significantly and blood tests showed an improved immune profile. The growth of the lung cancer was suppressed, and the patient is completely symptom-free. After completing radioimmunotherapy, the patient continues to live a life similar to that of a healthy person. This case suggests that radioimmunotherapy can be useful as an active treatment in patients who are not eligible for standard treatment.

## Introduction

In advanced lung adenocarcinoma with metastases, the current standard of care does not, in principle, include aggressive cancer treatment with surgery and radiotherapy. Therefore, a shift to palliative care is recommended when chemotherapy is ineffective or unsustainable. However, even if standard treatment is no longer possible, the patient's physical condition is not necessarily poor, in which case other treatment modalities can be used. In recent years, radioimmunotherapy, which combines radiotherapy and immunotherapy, has attracted attention. While conventional radiotherapy aims to destroy cancer cells, radiation in radioimmunotherapy aims to increase cancer immunogenicity. And the combined immunotherapy aims to eliminate cancer cells [1].

There are few reported cases of radioimmunotherapy and most of them involve the combination of radiotherapy with immune checkpoint inhibitors [1,2]. However, immune checkpoint inhibitors are associated with serious immune-related adverse events, the development of which is a major obstacle in the treatment of advanced cancer. On the other hand, cancer immuno-cell therapies using the patient's own immune cells, such as WT1 dendritic cell vaccine therapy (WT1-DC), utilize the patient's own cells and thus have few of the side effects seen with immune checkpoint inhibitors [3,4].

Here, we report a case of a patient with stage IV lung adenocarcinoma who was unable to continue chemotherapy, and who successfully reduced the size of his massive lung cancer by radioimmunotherapy, combining WT1-DC and radiotherapy at the same time, and achieved maintenance of good physical condition.

## Case presentation

**Figure 1:**
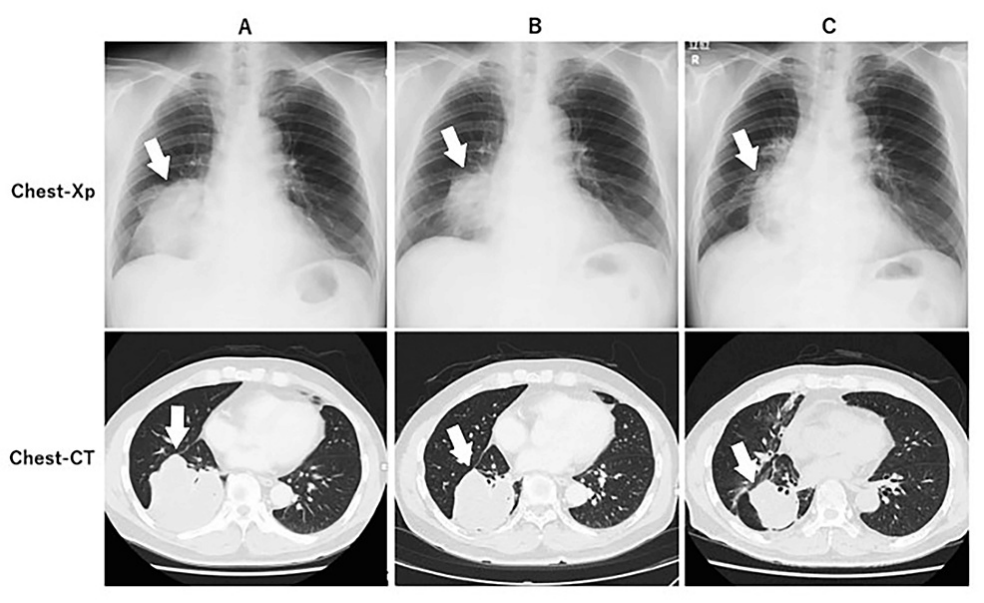
Lung cancer changes in chest Xp and CT A: - 16 days. B: 29 days. C: 130 days. Signs of radiation pneumonitis around the lung cancer. The white arrows indicate cancer.

We present a case of a male in his 60s who was healthy by nature. He was diagnosed with stage IIIB lung adenocarcinoma T4N1M0 following a cough, and CT showed a 9.1 x 9.0 cm diameter tumor in the S4 right lung (Figure 1A). The tumor was too large to be operated on. Genetic testing showed no indication for molecular targeted drugs, and chemotherapy with cisplatin 75 mg/m^2^, pemetrexed 500 mg/m^2^, and pembrolizumab 200 mg/body was started on -12 days, once every four weeks (the day radiotherapy was started was day 1).

**Figure 2:**
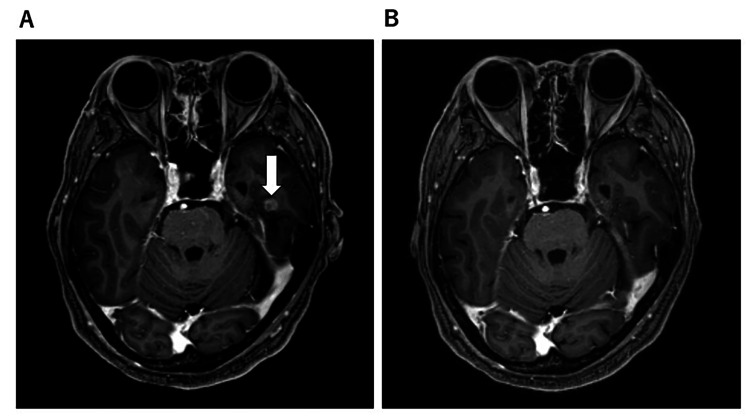
Brain metastases on brain MRI A: - 13 days. Metastatic lesion 1 cm in diameter in the medial part of the right temporal lobe. B: 88 days. Metastases have disappeared. The white arrow indicates cancer.

However, progressive weight loss due to pancytopenia and anorexia was expected to make it difficult to continue chemotherapy. Therefore, radioimmunotherapy was initiated. Intensity-modulated radiation therapy (IMRT) was initiated for right lung cancer and right pre-bronchial lymph node metastases with a planned dose of 60 Gy/30 fr. On the second day, a brain MRI showed a metastatic tumor 1 cm in diameter on the medial side of the right temporal lobe, and the patient was diagnosed as stage IV. Stereotactic radiotherapy was added to the brain metastases (Figure 2A).

**Figure 3:**
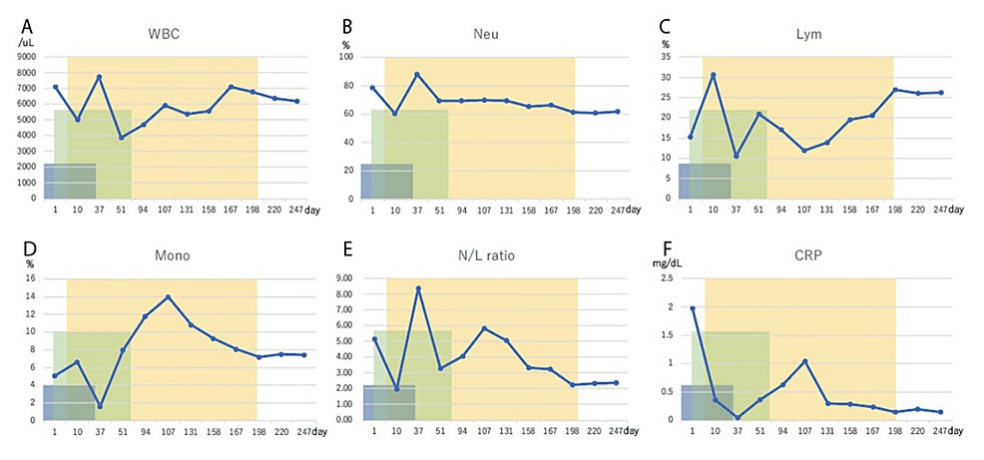
Changes in hematological indicators The blue areas indicate the periods during which cisplatin, pemetrexed, and pembrolizumab were administered. The green area is the period of radiotherapy. The yellow area is the period during which WT1-DC was performed. WBC: white blood cell, Neu: neutrophil, Lym: lymphocyte, Mono: monocyte, N/L: neutrophil/lymphocyte, CRP: C-reactive protein

**Figure 4:**
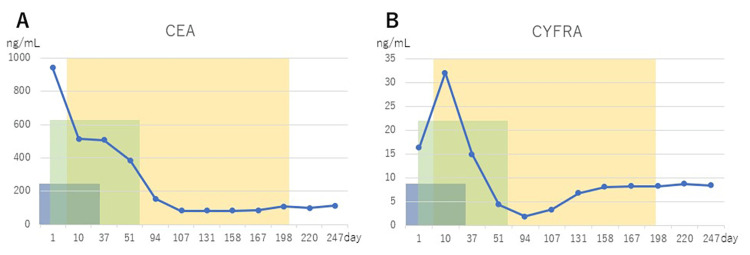
Changes in tumor markers The blue areas indicate the periods during which cisplatin, pemetrexed, and pembrolizumab were administered. The green area is the period of radiotherapy. The yellow area is the period during which WT1-DC was performed. CEA: carcinoembryonic antigen, CYFRA: cytokeratin 19 fragment

Parallel WT1-DC therapy was started on day 4. Monocytes were collected from the patient's blood and differentiated into mature dendritic cells over a period of two weeks at the cell processing center. WT1-DC was produced by making the dendritic cells recognize the common cancer antigen WT1 in culture. A total of 2.5 × 10^7^ WT1-DCs per dose were administered subcutaneously into the right and left para-inguinal lymph nodes. As of day 29, the lung cancer had shrunk (Figure 1B). However, due to side effects, all chemotherapy was stopped on day 44. During the course of radiotherapy, the blood levels of CRP, CEA, and CYFRA decreased while the white blood cell count, neutrophil count, lymphocyte count, monocyte count, and neutrophil/lymphocyte (N/L) ratio fluctuated sharply up and down (Figure 3, Figure 4).

**Figure 5:**
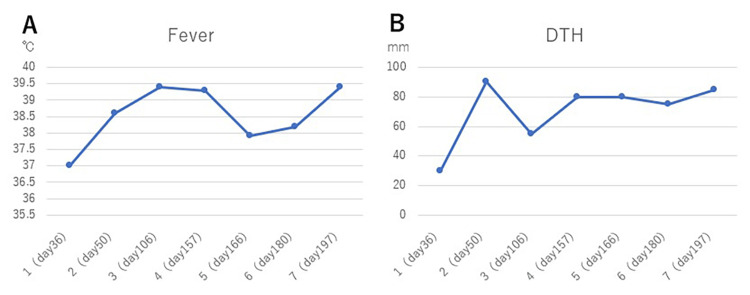
WT1-DC-mediated immune response Changes in fever and DTH magnitude following the administration of WT1-DC from the first to the seventh dose. Fever is the maximum body temperature within three days after administration. DTH is the largest mean of the short and long diameters of erythema within three days after administration. DTH: delayed type hypersensitivity

Radiation was completed on day 52. Brain MRI on day 88 showed that the metastases had disappeared (Figure 2B). On day 130, the lung cancer had shrunk further (Figure 1C). There were findings of radiation pneumonitis around the lung cancer but no clinical symptoms. WT-DC was administered seven times until day 197 and was completed. After the first WT1-DC dose, the patient had a fever of 37℃, but from the second dose onward, she had a high fever of 38.2-39.4℃ (Figure 5A). Delayed type hypersensitivity (DTH) is an index of the immune response of WT1 antigen-specific cytotoxic T cells. Intradermal administration of WT1 antigen to the medial forearm at the same time as the first dose of WT1-DC produced a DTH of 30 mm in diameter, whereas the second and subsequent doses of WT1-DC produced a strong DTH response of 55-90 mm (Figure 5B).

After the end of radiotherapy, during the period when WT1-DC was started, the patient showed an increase in lymphocyte count, a decrease in monocyte count, a decrease in N/L ratio, a decrease in CRP, and CEA and CYFRA also stabilized at low levels. WT1-DC was completed on day 197, but there was no further increase in lung cancer and the patient was in performance status 1 at day 247 with no clinical symptoms at all.

## Discussion

In this case, surgery and radiation were not possible according to the guidelines because the patient already had a large lung cancer when it was detected, and chemotherapy was the only option under standard treatment. However, the side effects of chemotherapy were so strong that it was necessary to discontinue chemotherapy. Generally, a shift to palliative care is recommended with the end of chemotherapy, but the patient wished to try radioimmunotherapy in this case.

During the course of IMRT at 60 Gy/30 fr for the primary right lung cancer tumor, the lung cancer shrank with a decrease and marked reduction in tumor markers. On the other hand, hematological assessment showed that the total white blood cell count, neutrophil count, lymphocyte count, monocyte count, N/L ratio, and CRP went up and down violently. These indices are known prognostic indicators in advanced cancer. Increased neutrophil counts, decreased lymphocyte counts, increased monocyte counts, elevated N/L ratio, and high CRP suggest a poor prognosis [5-9]. These sharp fluctuations in 'immune profile status' (IPS) are assumed to be due to changes in tumor-related inflammatory and anti-tumor immune responses induced by irradiation. Previous reports have shown that irradiation-induced DNA damage in tumor cells increases anti-tumor immune function through increased expression of HLAclass1, exposure of tumor antigens embedded within tumor cells to the cell membrane surface, or expression of neoantigens associated with DNA damage repair [10-12].

Radiation-induced damage to tumor DNA can also cause changes in tumor behaviors, which are reflected in the types and values of tumor markers. In the present case, irradiation caused a sharp decrease in CEA while CYFRA increased temporarily and then started to decrease. The clear difference in fluctuations depending on the type of tumor marker indicates a change in the nature of the tumor. The obvious shrinkage of the tumor along with the decrease in tumor markers during irradiation can be considered as a weakening of the viability of the tumor.

These changes in the nature of the tumor itself and its anti-tumor immunocompetence provide us with an excellent opportunity to intervene with therapy. In the tumor microenvironment, advanced cancers are protected from anti-tumor immunity in multiple ways, including expression of immune checkpoints, such as PD-1 and CTLA-4, reduced blood flow, increased extracellular matrix, and induction of immunosuppressive immune cells such as Tregs, myeloid-derived suppressor cells, and tumor-associated macrophages [10,11]. As these functions are established by the cancer, in many cases, the cancer is at least temporarily damaged by irradiation, and the immunosuppressive capacity of the cancer for anti-tumor immunity is reduced. Furthermore, increased cancer immunogenicity increases the activity of immune cells against the cancer, making the period during which the cancer is damaged by irradiation a very good opportunity for immunotherapy.

In this case, WT1-DC therapy was initiated three days after the start of radiation. WT1 is one of the cancer common antigens. According to a 2009 report by the National Cancer Institute in the USA, it ranked first among 75 common cancer antigens for an overall score of 9 items, including therapeutic efficacy, immunogenicity, cancer specificity, and expression [13]. Lung adenocarcinomas also express WT1 in more than 90% of cases [14]. In this patient, WT1 was used as the target antigen for the dendritic cell vaccine. The induction of WT1-specific cytotoxic T cells was considered sufficient, as the WT1-DC administration produced a high fever and DTH showed a clear erythematous response with a diameter of 55-90 mm. It is likely that irradiation increases the expression of HLA class 1 in cancer cells, thereby increasing the number of HLA classes presenting WT1.

Of note is the improvement in IPS during WT1-DC therapy. The blood test data from the third WT1-DC dose (day 106) onwards shows a marked increase in lymphocyte counts, a decrease in the N/L ratio, a decrease in monocyte counts, and a decrease in CRP values. As we have already reported, WT1-DC therapy has the effect of improving IPS [15]. Improved IPS indicates activation of anti-tumor immunity, and it is widely accepted by the immune checkpoint inhibitor trials that increased anti-tumor immunity is crucial for prolonged survival in cancer [16].

Combined radiotherapy and immunotherapy have been reported in various ways as radioimmunotherapy [17]. However, many radioimmunotherapies combine radiotherapy with immune checkpoint inhibitors. The effectiveness of the combination with immuno-cell therapy, as in the present case, has rarely been reported [18]. The main feature of autologous cell-based therapies is that they use the patient's own cells and therefore do not have the severe side effects observed with immune checkpoint inhibitors. Strong fever and large DTH are not side effects and should be interpreted as indicators of a main effect, indicating enhanced anti-tumor immunity.

In addition, given the risk that immune checkpoint inhibitors often produce severe life-threatening side effects, it is less risky and superior to utilize immuno-cell therapy with fewer side effects in patients whose physical functions are weakened by advanced cancer, suggesting the superiority of WT1-DCs in cancer treatment. On the other hand, the number of reported cases of radioimmunotherapy using WT1-DC is still small and needs to be validated through a series of ongoing research reports.

## Conclusions

Here, we report a case of stage 4 lung adenocarcinoma with brain metastases that could no longer be treated with chemotherapy, in which radioimmunotherapy successfully suppressed the growth of the tumor. It is considered that radioimmunotherapy is effective in controlling cancer even in advanced stages when standard treatment is no longer available and may contribute to prolonging survival. Immunotherapy using the patient's own immune cells, such as WT1-DC, can be safely administered without serious side effects, unlike immune checkpoint inhibitors. In advanced cancer, radioimmunotherapy, which can prolong survival with minimal risk, is at least a promising option after the end of standard treatment.
